# Assessment of left ventricular diastolic function in children with HIV/AIDS attending a tertiary health Facility in Enugu, Nigeria: a Doppler echocardiographic study

**DOI:** 10.1186/s12887-022-03719-y

**Published:** 2022-11-09

**Authors:** Ijeoma O. Arodiwe, Christopher B. Eke

**Affiliations:** 1 Department of Paediatrics, College of Medicine, University of Nigeria Enugu Campus, Nsukka, Nigeria; 2 Department of Paediatrics, University of Nigeria Teaching Hospital, Ituku- Ozalla, Enugu, Nigeria

**Keywords:** Diastolic function, Children, HIV/AIDS, Echocardiography

## Abstract

**Objective:**

To determine the prevalence and factors associated with left ventricular diastolic dysfunction in children with HIV/AIDS.

**Method:**

Echocardiographic studies were carried out in 90 children/adolescents aged 18 months to 14 years.

with HIV/AIDS and a healthy control group of 90 age and gender matched.

**Results:**

47.8% of the HIV/AIDS patients (subjects) had LVDD. This was more pronounced in the AIDS group (100%). The E/A ratio was 1.9 ± 0.56 in the HIV group, 2.09 ± 0.04 in the AIDS group, and 1.20 ± 0.39 in the control group (*p* = 0.04). The mean Left ventricular isovolumic relaxation time (IVRT) was 79.4 ± 20.12 in the HIV group, 110.4 ± 10.12 in the AIDS group and 89.22 ± 25.76 in the control group. (*p* = 0.04). Deceleration time (DT) was also lower in HIV carrier group compared to AIDS group, *p* = 0.02. A restrictive filling pattern was the most described; with 27 (36.5%) in the HIV group, 16 (100.0%) in the AIDS group and 2 (2.2%) in the control group. (*p* = 0.02). The impaired relaxation pattern, 3 (4.0%) seen in the HIV group only. Positive correlation exists between body surface area (BSA) and LVDD. Body surface area and younger age were the significant predictors (BSA: r = 0.425, *p* = 0.038 in HIV and r = 0.827, *p* = 0.042) of LVDD in the AIDS group.

**Conclusion:**

This study showed a high prevalence of LVDD in Nigerian children with HIV and AIDS. This justifies inclusion of echocardiographic studies in the policy care of children with HIV/AIDS in sub-Sahara Africa region.

## Synopsis

This study found the prevalence of left ventricular diastolic dysfunction (LVDD) in children with HIV/AIDS to be 47.8%. Body surface area was the best predictor of LVDD. Age correlated positively with LVDD in the AIDS group.

## Introduction

HIV/AIDS is one of the leading health problems in the world, especially in sub-Saharan Africa [1].

Recent studies have reported that HIV may exhibit cardiac tropism and that cardiac complications contribute significantly to morbidity and mortality in HIV infected children [2, 3].

As a result of availability of anti-retroviral (ARV) drugs and treatment of opportunistic infection, HIV infection has become a chronic disease.

ARV drugs may postpone the development of heart disease. Drugs like Zidovudine are cardiotoxic and have been linked with heart disease [3] The reported prevalence of LVDD in HIV infection from several studies in Europe and America varies from 2% to over 40% [4–6], with symptomatic heart failure developing in 6% [5, 7], most of whom have end-stage disease, AIDS [3, 6, 7]**.**

In sub-Saharan Africa where the burden of the disease is high, involvement of the heart by HIV.

cases has been recognized in clinical practice, however gap exists owing to the paucity of data on the subject [1, 3]**.**

The aim of the study was to evaluate LVDD and its associated factors in Nigerian children with HIV/AIDS, using Doppler echocardiography.

## Methods

This was a cross sectional descriptive study. Echocardiography was done using the recommendations of the American Society of Echocardiography [8, 9], to obtain measures of cardiac dimension and function, on 90 children with HIV/AIDS aged 18 months -14 years and their 90 age and gender matched healthy controls. Echocardiographic scanning and data collection were done by one of the contributors to the paper who is a is a certified paediatrics Cardiologist. The echocardiographic measurements were done according to the recommendations of the American Society of Echocardiography [8, 9] and three cycles were analyzed and average values were calculated and documented to minimize intra- observer variability. The subjects were positive HIV patients who were confirmed using serology and a clinical diagnosis of HIV infection according to CDC criteria and classified into 2 groups:

HIV-infected group with 74 (82.2%) and AIDS group with 16 (17.7%) patients respectively.

Sixty four cases (71.10%) were on highly active antiretroviral therapy (HAART), 10 (11.0%) on cotrimoxazole and anti-tuberculous therapy while 16 (17.70%) were on Cotrimoxazole for pneumocystis carinii jeroveci; pneumocystic carinii pneumocystic (PCP) prophylaxis. These two latter groups constitute the 26 (28.80%) patients that were not on HAART (HAART-Naive group). Among those on HAART, 61 (95.31%) were on 1st line drugs (a combination of Zidovudine (AZT), Nevirapine (NVP) and Lamivudine {3TC}) and 3 (4.69%) who were on 2nd line drugs, made up of a combination of Stavudine (d4t), lamivudine (3TC) and Lopinavir/ritonavir (LPV/r).

Ethical clearance was obtained from University of Nigeria Teaching Hospital (UNTH), Enugu Human Research Ethics Committee (HREC).

Subjects’ anthropometry, blood pressure and clinical profile were also obtained. The study was conducted from August 2017 to May 2019 at paediatric outpatients cardiology and HIV/AIDS clinics of UNTH. The latter is an outpatient follow-up clinic of children infected with HIV/AID. The clinic was supported then by the United States President’s Emergency Plan for AIDS Relief (PEPFAR). It provides free guideline - based comprehensive paediatric HIV/AIDS care. This includes prevention, treatment, and follow-up care.

### Echocardiographic studies

The following variables and its defining criteria mean (sd) were measured: left ventricular posterior wall thickness in diastole for calculating left ventricular mass (Devereux’s formula) [10], isovolumic relaxation time (IVRT) = 71 [11] ms, mitral deceleration time (DT) 199 (32) ms. Deceleration time (DT) is the time interval from the peak of the E-wave to its projected baseline. The E-wave deceleration time is normally between 150 ms and 240 ms. The deceleration time indicates the duration for equalizing the pressure difference between the left atrium and the left ventricle, early peak (VE) = 0.86 (0.16) ms; late peak (VA) = 0.56 (0.13) ms; VE/VA ratio = 1.6 (0.4); LV diastolic dysfunction was present if: E/A ratio < 1, IVRT > 100 m/s and DT > 220 m/s (impaired relaxation pattern); pseudo- normalization resembling the normal configuration with respect to the mitral inflow but with low DT and the restrictive pattern is present when E/A ratio > 2, IVRT< 70 m/s and DT < 150 m/s [9]**.**

### Statistical analysis

Statistical package for Social Sciences version 20.0 was used to enter and analyze the data. Descriptive statistics for continuous variables were expressed as mean (SD). Qualitative variables were presented as percentages or frequencies. Test of association was determined using one way ANOVA or chi-square test as appropriate. A *p* value < 0.05 was considered statistically significant. Pearson’s correlation and regression analysis were used to assess the relations between LVDD as dependent variable and its independent variables.

## Results

Ninety patients were recruited for the study, 74 were HIV infected and 16 had AIDS.

The characteristics of the study participants are shown in Table 1.

**Table 1:** Characteristics of the study participants

Characteristics	HIV infection	AIDS	controls	F/χ^**2**^	p – value
	***N*** = 74	***n*** = 16	***n*** = 90		
Mean age (years)	8.15 ± 3.08	7.9 ± 2.07	8.3 ± 3.04	0.14	0.87
Gender M/F	38 /36	9/7	49/41	0.654	0.06
Mean weight (kg)	14.43 ± 9.67	10.22 ± 6.07	22.4 ± 9.42	21.30	< 0.001
Mean height (cm)	108.1 ± 20.9	95.7 ± 15.3	114.7 ± 21.8	6.28	0.002
Mean BMI for age	18.3 ± 2	16.4 ± 2	22 ± 3.1	337.81	< 0.001
Mean RR/min	29 ± 5	32 ± 6	26 ± 5	13.12	< 0.001
Mean HR/min	103 ± 18	120 ± 20	92 ± 13	25.05	< 0.001
Mean BP (mmHg)	89 ± 8	81 ± 12	85 ± 12	5.14	< 0.007
Mean ESR (mm/1st hr)	31 ± 10.6	67 ± 12.4	6.3 ± 2.4	486.40	< 0.001
Mean CD4+ (cells/mm^3^)	1486.6 ± 158.6	504.6 ± 300.3	1786.6 ± 1582.6	8.93	< 0.001
CD4+ (cells/mm^3^)					
≤ 1499, n(%)	6(8.1)	15(93)	30(3.3)	5.6	0.01
≥ 1500, n(%)	68(92)	1.1(6.9)	87(96)	4.54	0.05

Key: *BMI* Body Mass Index, *M* Male, *F* Female, *RR* Respiratory Rate, *HR* Heart Rate, *BP* Blood Pressure, *HB* Haemoglobin, *ESR* Erythrocyte Sedimentation Rate.

The mean weight, height and body mass index, BMI for age were significantly higher in the controls.

than HIV and AIDS group. The mean RR, HR, BP and ESR were significantly higher in the AIDS group.

The AIDS group also had a higher number of persons with severely depressed CD4+ cell counts, as shown in Table 1.

LVDD was present in 43 (47.8%) of the 90 children with HIV and AIDS. However, prevalence of LVDD was significantly higher in AIDS group (100%), compared with patients with HIV – infection (36.5%) and controls (2.2%), (*p* = 0.03). Mean E/A ratio, IVRT and DT were significantly higher in the AIDS group, as shown in Table 2.

**Table 2:** Echocardiographic characteristics of the study participants

Characteristics	HIV infection N = 74	AIDS ***n*** = 16	Control ***n*** = 90	F/ χ^**2**^	p
Prevalence of LVDD. N (%)	27(36.5)	16(100)	2(2.2)	1.23	0.03
Mean LVMI(g/m^2^)	90.4 ± 25.3	89.4 ± 25.1	74.5 ± 23.2	9.47	0.001
Mean E/A ratio	1.9 ± 0.56	2.09 ± 0.46	1.20 ± 0.39	2.03	0.01
Mean IVRT(m/s)	79.4 ± 20.12	110.4 ± 10.12	89.22 ± 25.76	2.11	0.04
Mean DT(m/s)	184.66 ± 76.27	230.66 ± 56.27	174.10 ± 38.24	0.37	0.02
Mean LVDd(mm)	5.96 ± 0.6	6.75 ± 0.6	3.75 ± 0.72	0.70	0.02
Mean LVESd(mm)	2.65 ± 0.18	3.75 ± 0.36	2.69 ± 0.18	3.30	0.01

Key: *LVMI* Left Ventricular Mass Index, *E/A* ratio Early/Atrial Mitral Inflow Velocities, *IVRT* Isovolumic Relaxation Time, *DT* Deceleration Time, *LVEDd* Left Ventricular End Diastolic dimension, *LVESd* Left Ventricular End Systolic dimension, *LVDD* Left Ventricular Dystolic Dysfunction

**Fig. 1:**
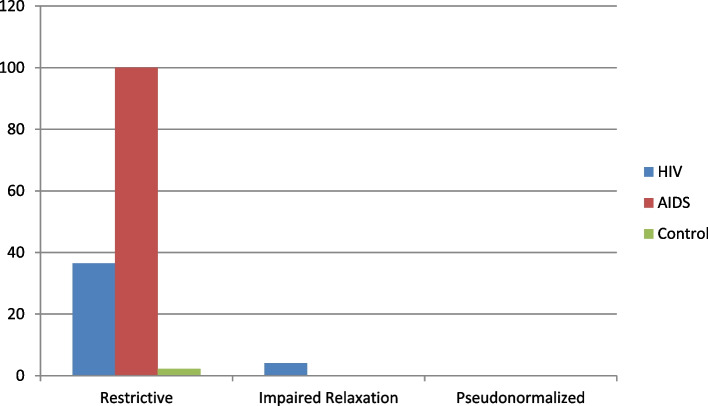
Clustered column showing patterns of LVDD in HIV, AIDS and Control groups

A restrictive pattern was the most described in the groups as shown in fig. 1, showing 36.5% in the HIV group, 100 and 2.2% in the AIDS and control groups respectively. The impaired relaxation patter was observed among the HIV group occurring in 4.1% of them. None of the groups (HIV, AIDS, or control) had the pseudonormalization of LV dysfunction.

The body surface area (BSA) correlated positively with LVDD; r = 0.425, p = 0.038 in HIV and r = 0.827, *p* = 0.042 in AIDS. Logistic regression showed BSA and age as the only predictors of LVDD in.

both group, as shown in Table 3.

**Table 3:** Correlation of independent variables with diastolic function in HIV/AIDS patients and Controls

Parameters	HIV infection		AIDS		Control	
	r	p	r	p	r	p
Age	0.320	0.127	0.874	**0.023**	0.212	0.521
BMI	0.104	0.628	−0.199	0.706	0.120	0.102
Pulse rate	− 0.301	0.152	−0.254	0.627	0.401	0.092
BSA	**0.425**	**0.038**	**0.827**	**0.042**	0.031	0.723
LVMI	−0.013	0.951	0.00	0.951	0.192	0.821
HB	−0.193	0.365	−0.682	0.135	0.252	0.601
CD4 cell count	−0.392	0.058	−0.266	0.610	0.313	0.161

Key: *BMI* Body Mass Index, *BSA* Body Surface Area, *LVMI* Left Ventricular Mass Index, *HB* Haemoglobin

## Discussion

The study shows that HIV/AIDS infection is associated with both increased LV mass index and diastolic dysfunction. LVDD was found in 47.8% of the children as shown in Table 2. Ige et al. [12], in Jos, noted 30.7%.

Also Lipshultz, et al. [13], in Boston reported 39%. These differences in prevalence might be type of study design and possible some racial or genetic predisposition. The most common significant variation was an increased atrial contribution to ventricular filling in the HIV- infected and AIDS group denoting restrictive pattern as shown in fig. 1. This has also been reported by various workers [10, 14]**.** The reversal of filling pattern is likely due to left ventricular hypertrophy, especially as the HIV-infected subjects in this study had significantly elevated left ventricular mass index. Some authors noted that left ventricular diastolic impairment is an important pathophysiologic mechanism reflecting the symptoms of congestive heart failure [10, 14]**.** This was not found in this index study. It may be due to heart failure, being a late manifestation of diastolic dysfunction. The significance of a high prevalence of LVDD relates to its association with morbidity and mortality [11].

The mean weight in the AIDS group was significantly lower than the controls, as shown in Table 1. Weight loss has been known to be associated with decrease in left ventricular mass, volume and function [15, 16]**.**

However the presence of increased LVMI indicating LVH in the patients may be an indication that some other factors, such as proto-oncogene activation can modulate the hypertrophic response [17]**.** This may have counteracted the usual effects of malnutrition on cardiac muscles. Body surface area (BSA) and total white cell count (WBC) correlated significantly with LVDD in the HIV group, the former positively and the later negatively, as shown in Table 3. In the AIDS group, age and BSA correlated with LVDD. These findings are difficult to explain. Positive correlation of BSA with LVDD suggests that the higher the body surface area, the higher the prevalence of LVDD. The relationship between BSA and LVDD dysfunction. Could be simply reflect older age and/or more years with chronic infection, Our finding is at variance with Lipshultz et al. [13] and Lobato, et al. [18] who noted the presence of HIV encephalopathy as predictor of LVDD in HIV/AIDS infection. This difference may be due to their study population, which were only perinatally acquired HIV infected children. In the HIV group, the lower the total WBC count, the more likely LVDD was present. This agrees with the report of Herskowitz et al. [19], who found a median CD4+ cell count of 30/μl compared 187/μl in those without LVDD [18] Lower CD4+ cell count is a marker of terminal disease associated with HIV cardiomyopathy and a rapid course of disease progression with end organ involvement [20].

Age of the patients correlated with LVDD in the AIDS group. This suggests that the older the patient, the higher the prevalence of LVDD. This could be explained by the fact that the virus and its effects on the heart muscle and other effects have had more time to occur. Some other studies showed that higher LVM which predisposes to LVDD was more common in older HIV-infected children [13, 20]**.**

Increased LVM underlying LVDD may be related to an increase in sympathetic tone, a manifestation of autonomic dysfunction. This in turn raises the concentration of catecholamine eventually leading to myocardial hypertrophy. Other risk factors predisposing to LVDD like tachycardia, blood pressure, haemoglobin level, and stage of infection appear not to correlate with LVDD in this study. This was also the observation of Ige et al. [12]

The methods used in this study to assess LVDD are limited by the confounding effects of physiologic variables including LV relaxation, and atrial filling pressure. The new techniques; tissue Doppler imaging (TDI) [21], using the lateral annular early diastolic peak velocity could have also been used.

Indeed, the relationship between mitral annular velocities from tissue Doppler imaging (TDI) (E’ and A’) and mitral inflow velocities (E and A) from Doppler echocardiography (DE) would provide additional information about LV filling and diastolic function. The dearth of access to this newer technology of LV diastolic function and lack of assessment of LA size are limitations of this study.

## Conclusion

This study showed that LVDD is common in children with HIV/AIDS who are asymptomatic of heart failure. The echocardiographic indices of LV diastolic function and the prevalence of LVDD were significantly.higher in the HIV/AIDS group compared to controls. Body surface area was the only predictor of LVDD.Total WBC correlated with LVDD in the HIV group while age in those with AIDS. This underscores.the need for integrating routine baseline and periodic cardiovascular assessment using echocardiography, especially in selected patients.

## Data Availability

The data and materials used for the article are available with the author and can be made available on minimal request. All requests for access to the data from this study should be addressed to the corresponding author: Prof Christopher B. Eke via email: Christopher.eke@unn.edu.ng.
